# *Microorganisms* 2018 Best Paper Award

**DOI:** 10.3390/microorganisms6040120

**Published:** 2018-11-28

**Authors:** Microorganisms Editorial Office

**Affiliations:** MDPI, St. Alban-Anlage 66, 4052 Basel, Switzerland; microorganisms@mdpi.com

*Microorganisms* is instituting an annual award to recognize the outstanding papers published in the journal.

We are pleased to announce the “*Microorganisms* Best Paper Award” for 2018. Nominations, chosen from all papers published in 2016 and 2017, were made by the Editorial Board. Following review by the Editorial Board, the top-voted research article and the top-voted review, which have won the “*Microorganisms* Best Paper Award” for 2018 are, as follows, in no particular order:

## Research Article Award:

**Extremophiles in an Antarctic Marine Ecosystem**

Iain Dickinson, William Goodall-Copestake, Michael A.S. Thorne, Thomas Schlitt, Maria L. Ávila-Jiménez and David A. Pearce

*Microorganisms***2016**, *4*(1), 8; doi:10.3390/microorganisms4010008

Available online: https://www.mdpi.com/2076-2607/4/1/8


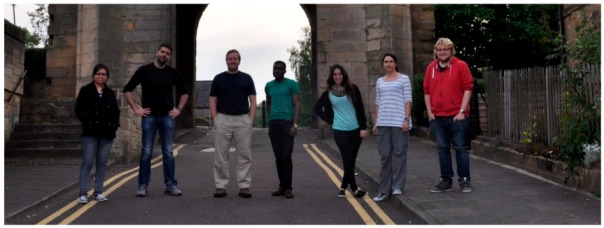


Our group has been studying the diversity, community structure, stability, and distribution of microorganisms in the Polar regions and other extreme environments for nearly 20 years. During this time, it has become clear that a biogeography of microorganisms exists across these environments. With this distribution of taxonomic diversity, it seemed logical that there might also be a distribution of functional diversity. This manuscript was one of the first steps in investigating that biogeographic distribution of functional diversity. In coping with environmental extremes, microorganisms have developed a range of adaptations, and so we suspected that these environments might also be a rich source of novel genetic material that might be exploited for bioprospecting applications. In this study, we focused on the community within the boundary region located between the Polar Front and the Southern Antarctic Circumpolar Current in the Southern Ocean, to explore the potential of metagenomic approaches as a tool for bioprospecting in the search for novel functional activity based on targeted sampling efforts. In the study, we found a proportion of genes related to secondary metabolism of potential interest in bioprospecting. We also found that extremophiles, although rare by comparison, belonged to a range of genera, hence, they represented interesting targets from which to identify rare or novel functions. We also suggested that future shifts in environmental conditions favoring more cosmopolitan groups could have an unpredictable effect on microbial diversity and function in the Southern Ocean, perhaps excluding the rarer extremophiles.

## Review Paper Award:

The Gut Microbiome Feelings of the Brain: A Perspective for Non-Microbiologists

Aaron Lerner, Sandra Neidhöfer and Torsten Matthias

*Microorganisms***2017**, *5*(4), 66; doi:10.3390/microorganisms5040066

Available online: https://www.mdpi.com/2076-2607/5/4/66


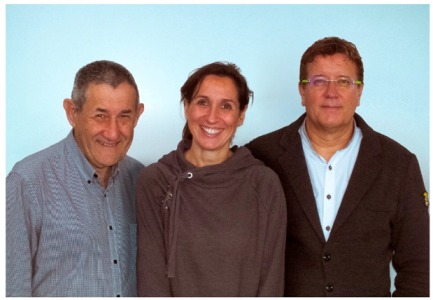


The brain–gut connection has gained recognition as a major contributor to human health, but the gut–brain axis, which is essential for daily life and contributes to human diseases, merits equal attention.

Many reviews have focused on the top-down, brain to gut axis, however, the present review expands and updates from the bottom-up, namely, the gut to brain axis. This involves multiple environmental factors, gut eco-events, and two major players, nutrients and the second brain—the microbiome. The combined notion of nutrition, microbiota, mucosal, immune, endocrine, neuronal, and brain circuitries are too complicated, but contain the pure truth. In reality, the two opposite directions refer to a bidirectional communication that mutually affects and depends on the other. It encompasses multiple intricate systems that were shaped during human evolution to maintain homeostasis and protect the body against detrimental factors, establishing symbiotic relations between bugs and us. Several routes are suggested to deliver the informatics knowledge from the intestinal tract to the brain: neuroanatomical, neuroendocrine, immune, macrobiotic, and the gut and brain barrier pathways. Afferent vagus routes play an essential role in bringing the lower signals up to the brain. The balanced functioning of the gut–brain axis depends on the normal functional activity of the vagal nerve. The present review reflects a non-infectious, gastroenterological view, and, as such, concentrates more on the enteric eco-events than on the very complicated central nervous system, which is a never-ending labyrinth.

We believe that the two exceptional papers are valuable contributions to *Microorganisms* and the scientific research field. On behalf of the *Microorganisms* Editorial Board, we would like to congratulate their teams for their excellent work. A certificate will be given to each of them.

We would like to take this opportunity to thank all the nominated research groups of the above exceptional papers for their contributions to the *Microorganisms*, and thank the *Microorganisms* Editorial Board for voting and helping with this “Best Paper Award”.

The Editorial Board and Editorial Staff at *Microorganisms* is committed to meeting the needs of the scientific community by providing useful and timely reviews of all manuscripts submitted, and providing an open access journal for your results. Please consider submitting your work to *Microorganisms*, and we look forward to announcing your paper as a *Microorganisms* Best Paper in the future.

## Prize Awarding Committee

*Microorganisms* Editorial Board.

